# Dynamic detection of electron spin accumulation in ferromagnet–semiconductor devices by ferromagnetic resonance

**DOI:** 10.1038/ncomms10296

**Published:** 2016-01-18

**Authors:** Changjiang Liu, Sahil J. Patel, Timothy A. Peterson, Chad C. Geppert, Kevin D. Christie, Gordon Stecklein, Chris J. Palmstrøm, Paul A. Crowell

**Affiliations:** 1 School of Physics and Astronomy, University of Minnesota, Minneapolis, Minnesota 55455, USA; 2 Department of Materials, University of California, Santa Barbara, California 93106, USA; 3 Department of Electrical and Computer Engineering, University of California, Santa Barbara, California 93106, USA

## Abstract

A distinguishing feature of spin accumulation in ferromagnet–semiconductor devices is its precession in a magnetic field. This is the basis for detection techniques such as the Hanle effect, but these approaches become ineffective as the spin lifetime in the semiconductor decreases. For this reason, no electrical Hanle measurement has been demonstrated in GaAs at room temperature. We show here that by forcing the magnetization in the ferromagnet to precess at resonance instead of relying only on the Larmor precession of the spin accumulation in the semiconductor, an electrically generated spin accumulation can be detected up to 300 K. The injection bias and temperature dependence of the measured spin signal agree with those obtained using traditional methods. We further show that this approach enables a measurement of short spin lifetimes (<100 ps), a regime that is not accessible in semiconductors using traditional Hanle techniques.

## Abstract (web summary)

In semiconductor spintronic devices, Hanle precession allows for electrical detection of spin accumulation however it is inhibited at room temperature in GaAs by magnetic-field effects. Here, the authors present an alternative method for detecting spin accumulation based on ferromagnetic resonance.

## Graphical abstract

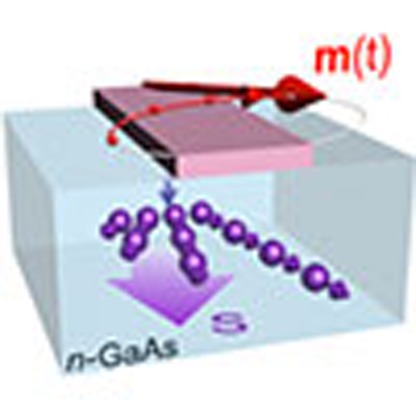

An effective means to electrically detect electron spin accumulation in a semiconductor (SC) is essential for the development of SC spintronics^1^,^2^. A common detection method, known as the Hanle effect^3^, relies on the precession of the spin accumulation in the SC by an external magnetic field. This approach has been employed in both the non-local^4^,^5^ and the local 3-terminal (3T)^6^ measurement geometries. As the spin lifetime decreases at higher temperatures, a significantly higher magnetic field is required in order for the Hanle effect to dephase the spin accumulation^7^. The ordinary magnetoresistance in SCs in such large fields produces large quadratic backgrounds^6^ that mask any truly spin-dependent effects. As a result, no electrical Hanle measurement has been demonstrated in *n*-GaAs at room temperature. Although the 3T technique has recently been widely used to study spin transport in new material systems^8^,^9^,^10^ at higher temperatures, it has been shown that the 3T Hanle measurement is sensitive to a variety of magnetic field-assisted phenomena^11^,^12^,^13^, which often cannot be separated from the Hanle effect.

We introduce here a detection technique that utilizes the precession of the magnetization under ferromagnetic resonance (FMR) to dynamically detect the spin accumulation in a SC. The approach is similar in some aspects to a Hanle measurement, in that the observed signal corresponds to a suppression of the component of the spin accumulation parallel to the magnetization. In this case, however, the suppression is due to the onset of FMR (precession of the magnetization in the source and detector) rather than precession of the spin accumulation in the SC. The narrow FMR linewidth and the sensitivity to both the precession of the magnetization in the ferromagnet (FM) as well as the dynamics of the spin accumulation in the SC provide an immunity to the field-dependent backgrounds that affect Hanle measurements, particularly in the 3T case. At low temperatures, the Larmor precession of the spin accumulation in the SC can be detected by an FMR measurement in an oblique magnetic field. We verify the effectiveness of this approach by comparing the bias and temperature dependence of the measured spin signal to results obtained on the same heterostructures using traditional spin detection methods. Finally, we show that this FMR-based spin detection technique enables one to determine the spin relaxation rate in GaAs at room temperature, at which an ordinary Hanle curve cannot be measured.

## Results

### Electrical detection of spin accumulation by a FM

The devices used in this electrical spin injection and detection experiment consist of FM–SC heterostructures. A spin current across the FM/SC interface is generated by a tunnelling charge current^14^,^15^, leading to a spin accumulation^16^, which has previously been detected potentiometrically^5^,^17^ or by using a spin filter to detect the polarized current^18^. In the potentiometric approach, the voltage detected by a FM contact due to spin accumulation can be expressed as^5^


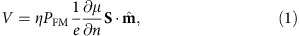


where *η* is the spin detection efficiency, *P*_FM_ is the spin polarization at the Fermi level in the FM, *e* is the electron charge and *μ* and *n* are the chemical potential and number density of electrons in the SC, respectively. The spin accumulation **S** in the SC has a magnitude defined as 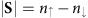 where *n*_↑_ and *n*_↓_ refer to the number density of spin-up and spin-down electrons, and 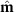 is the unit vector of the magnetization **m** in the FM. The dot product in equation (1) accounts for the projection of the spin accumulation, which must be averaged over the area of the contact, on the magnetization. In a Hanle measurement, one applies a magnetic field perpendicular to 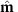 to precess and dephase the injected spins, thus reducing the magnitude and changing the orientation of **S**. In our experiment, we force the magnetization of the FM to precess instead of the spin accumulation in the SC. We inject spins from the FM and simultaneously drive the magnetization in the FM to precess by FMR. During this process, 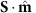 also changes, which allows for detection of the spin accumulation. As will be explained in detail in the modelling section, the important timescales in this experiment are the FMR-precession period and the electron spin lifetime *τ*_s_ in the SC. Together, these determine the steady-state value of 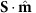, which changes at resonance.

### Experimental set-up and room temperature measurement

**Figure 1:**
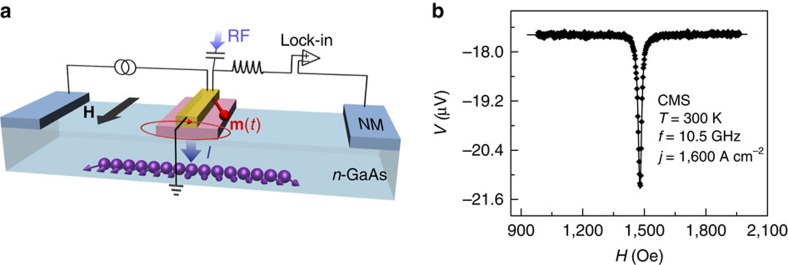
Schematic diagram of the experiment and the spin accumulation measured by FMR. (**a**) A schematic diagram of the FMR-spin detection experiment. The red contact in the middle of the device is ferromagnetic. On top of the ferromagnet is the microwave stripline (shown in yellow). The two remote contacts (shown in blue) are ohmic and are fabricated from non-magnetic Cu–Ge. **H** and **m**(*t*) represent the applied magnetic field and the precessing magnetization of the FM, respectively. *I* is the applied interface bias current, which generates a spin accumulation in *n*-GaAs. Spins (shown in purple) injected by the precessing magnetization at different times have different orientations, and they diffuse in the *n*-GaAs channel. (**b**) Spin signal measured by FMR as a decrease in voltage at room temperature. The solid line is a Lorentzian fit to the data. CMS refers to the sample with Co_2_MnSi as the FM.

Figure 1a is a schematic diagram of the FMR-spin detection experiment. The devices used in our experiment are fabricated from epitaxial FM/*n*-GaAs (100) heterostructures. Recently, the utilization of ferromagnetic Heusler alloys^19^ has greatly improved the spin injection efficiency into GaAs (refs 20, 21). In our experiment, we use the Heusler alloys Co_2_MnSi and Co_2_FeSi as spin injectors. The FM film is 5 nm thick with lateral dimensions of 5 μm × 50 μm. The *n*-GaAs is doped with a carrier concentration of *n*=3 × 10^16 ^cm^−3^. The junction region consists of a highly doped *n*^*+*^ (5 × 10^18 ^cm^−3^) layer to create a Schottky tunnel barrier^14^, allowing for a high spin injection efficiency^22^,^23^. A 120 nm-thick gold wire, which forms a microwave stripline, is deposited directly on top of the FM contact. The microwave Oersted field from the current passing through the stripline drives the resonance. The microwave frequency used in the experiment ranges from 5 to 20 GHz. The applied d.c. magnetic field is in the sample plane as indicated by **H** in Fig. 1a. A forward bias current across the FM/SC interface injects a spin current into the *n*-GaAs (ref. 24). The microwave field is modulated at low frequency (100 Hz) and a lock-in amplifier synchronized with the modulation frequency measures the difference in interface voltage with microwaves on and off.

When the bias current generates a spin accumulation in the *n*-GaAs and the magnetization is driven on resonance, a dip in the three-terminal voltage is observed. The raw data in Fig. 1b show the resonance observed at room temperature. The resonance field measured as a function of the microwave frequency agrees with the FMR spectrum as calculated from Kittel's formula^25^ using the anisotropy and magnetization of each sample. The resonant field as a function of frequency is shown in Supplementary Fig. 1, where it is compared with the calculations of Supplementary Note 1. The non-zero background voltage in Fig. 1b is due to the nonlinear IV characteristic of the FM/*n*-GaAs Schottky contact^26^, which rectifies the microwave current generated in the device. We see that the interface voltage decreases on resonance, which, as will be explained in detail below, is the result of the decrease in the projection of **S** on 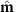. In previous work, we found that the tunnelling anisotropic magnetoresistance (TAMR) effect^27^,^28^ at the FM/SC interface also produces a change in the interface voltage at FMR^29^. The size of the TAMR effect depends on the bias voltage across the FM/SC interface. When a high forward bias current is applied to the sample, generating a spin accumulation in the SC, the FMR signal due to the TAMR effect is much smaller than that produced by the spin accumulation^29^. In the data analysis below, the small contribution to the FMR signal from the TAMR effect has been removed.

Although this experiment is designed to detect the spin accumulation generated by the bias current, there are other effects in addition to TAMR that could yield a resonant peak in the d.c. voltage measured across the junction. First, microwave currents flowing in the FM itself can be rectified by the ordinary anisotropic magnetoresistance effect^30^. Although the design of our devices minimizes such a contribution, rectification by anisotropic magnetoresistance also produces a distinct (and orientation-dependent) lineshape^31^, which we do not observe. We also consider the possibility that a spin accumulation can be created by spin-pumping from the FM under FMR^32^. Although spin-pumping signatures have been observed in FM–SC heterostructures^33^,^34^, we do not observe a resonant peak at zero d.c. bias. This is consistent with the fact that, unlike the samples used in the previous spin-pumping experiments^33^,^34^, the Schottky barriers used in our epitaxial FM/*n*-GaAs heterostructures are highly rectifying and not transparent at zero bias. If present, any contribution from spin-pumping is negligible in comparison to the spin accumulation generated by the d.c. bias current. Furthermore, the sign and shape of the FMR peak are independent of the direction of the magnetization, which excludes any significant contribution from inverse spin Hall voltages associated with spin pumping.

### Detecting the Larmor precession

**Figure 2:**
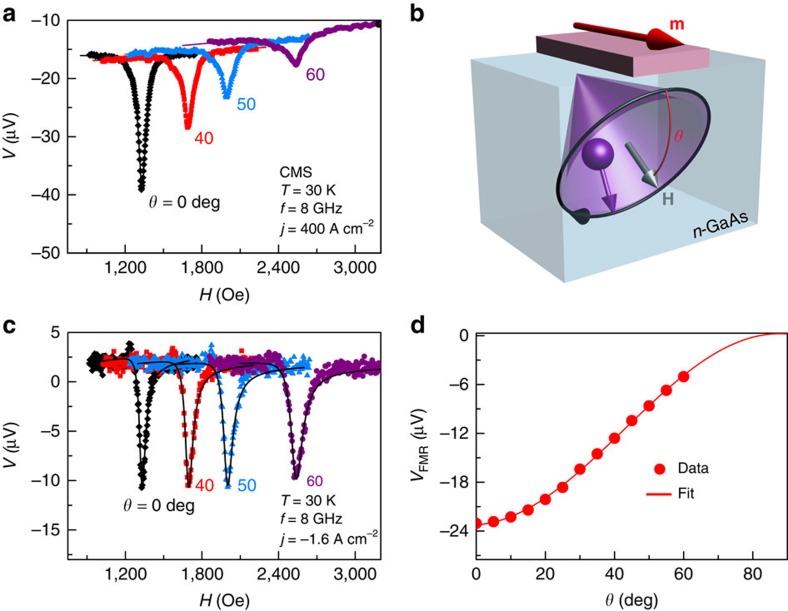
Experimental results in the oblique configuration and illustration of the Larmor precession. (**a**) The FMR signal measured at different out-of-plane angles of the applied field when a spin accumulation exists in the *n*-GaAs. Note the decrease in the signal amplitude with increasing angle. (**b**) The applied magnetic field **H**, represented by the grey arrow, is tilted out-of-plane by an angle *θ*. The injected spins precess about **H**, and their directions are uniformly distributed on a cone when 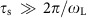, as is the case at 30 K. (**c**) FMR measured under reverse bias, at which only the TAMR effect is present. The out-of-plane angles are the same as in **a**. In this case the amplitude is nearly independent of angle. The solid lines show the predicted FMR signals, which are obtained by calculating the magnetic susceptibilities with the magnetization tilted by the applied magnetic field. (**d**) Angle dependence of the magnitude of the spin accumulation signal measured by FMR (**a**) and a fit based on the Larmor precession model. The raw data in **a**,**c** are plotted as a function of the total magnetic field, and so the resonance field positions and the widths of the peaks scale as 1/cos*θ* relative to the in-plane (*θ*=0) case.

A distinguishing feature of spin is the Larmor precession about an applied magnetic field. The precession frequency is 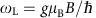, where *μ*_B_ is the Bohr magneton, the *g* factor is −0.44 for electrons in GaAs (ref. 35) and *ħ* is the reduced Planck constant. We expect that the Larmor precession in the SC will influence the spin accumulation measured by FMR when *ω*_L_ is larger than the spin relaxation rate. In these samples, this condition can be achieved at low temperatures (30 K), at which the spin lifetime for this doping is longer than 10 ns (ref. 36), while the Larmor precession period is ∼1 ns for a magnetic field of 2,000 Oe. We tilt the magnetic field out of the sample plane such that the perpendicular component of the magnetic field is still much smaller than the magnetic anisotropy field of the FM film. This allows the magnetization to remain nearly in the sample plane, while the spins in the SC precess about the oblique magnetic field. In the steady state, the spin accumulation can be viewed as an ensemble of spins injected at different times^3^. When 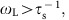 as is the case at 30 K, the orientations of electron spins are therefore distributed uniformly on the precession cone, which is depicted in purple in Fig. 2b. In other words, the transverse component of the spin accumulation is completely dephased, and there is no component of the steady-state spin accumulation perpendicular to the applied field.

Figure 2a shows that the FMR-spin accumulation signal decreases significantly as the out-of-plane angle *θ* of the magnetic field increases. To verify that this change is not simply due to the magnetization dynamics of the FM, we confirm that the amplitude of the precession cone angle in the FM does not change significantly with increasing angle by applying a reverse bias across the FM/*n*-GaAs interface. Under reverse bias, there is negligible spin accumulation generated in the *n*-GaAs, as we have verified in traditional non-local spin valve measurements, and the interface voltage at resonance is due only to the TAMR. The TAMR can in turn be related very simply to the geometry of the precessing magnetization in the FM, which is discussed in Supplementary Note 2. Figure 2c shows almost no change in the size of the voltage peak when the field is tilted out of plane, and hence we conclude that the amplitude of the precession cone angle in the FM is nearly constant. This is consistent with calculations of the susceptibility at different angles, as discussed in Supplementary Note 3 (see also Supplementary Fig. 2). The solid curves in Fig. 2c show the FMR signal calculated for different angles by using the in-plane data and scaling by the susceptibility calculated for each field orientation.

The spin accumulation detected by the FM is proportional to 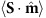, where the brackets indicate the time average in the steady state. Because the transverse spin is completely dephased under the conditions being considered, the magnitude of the spin accumulation is reduced by a factor of cos*θ* relative to its magnitude *S*_0_ when the field is in the sample plane. As a result, 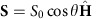, where 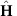 is a unit vector parallel to the magnetic field. Because the detected voltage is proportional to 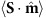 and none of the prefactors in equation (1) depends on angle, the spin accumulation signal at resonance should therefore vary as cos^2^*θ*. Figure 2d shows the amplitude of the resonant signal *V*_FMR_ (the magnitudes of the negative peaks in Fig. 2a) as a function of *θ*. The solid line is a fit to a function proportional to cos^2^*θ*, and therefore the behaviour expected for a spin accumulation signal is observed.

### Comparison with other measurement techniques

**Figure 3:**
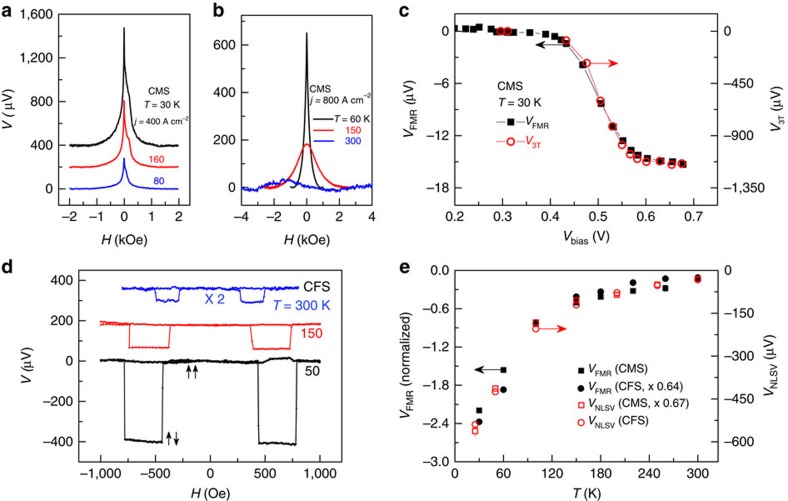
Bias and temperature dependence measured by traditional methods and FMR. (**a**) 3T data measured as a function of perpendicular field at 30 K for different injection bias currents. (**b**) 3T signal measured at different temperatures for a fixed bias current. The spin signal cannot be resolved using the Hanle measurement at 300 K. (**c**) Magnitude of the FMR peak, *V*_FMR_ (black squares, left axis), and *V*_3T_ (red circles, right axis) plotted as a function of injection bias voltage at 30 K. Note that the two sets of measurements differ by only a single bias-independent scale factor. (**d**) Non-local spin valve measurements at different temperatures, obtained at a source–detector separation of 250 nm. The 300-K data are multiplied by a factor of 2 for clarity. The arrows indicate the relative orientations of the magnetization in the source and detector FM. In **a**,**b**,**d**, a second-order background has been subtracted from the raw data. Curves in **a**,**d** are offset for clarity. (**e**) *V*_FMR_ (black solid symbols, left axis), normalized by the precession amplitude and *V*_NLSV_ (red open symbols, right axis) plotted as a function of temperature. CMS and CFS refer to samples with Co_2_MnSi and Co_2_FeSi as the FM, respectively.

To obtain further evidence that the observed FMR signal is due to the spin accumulation in the *n*-GaAs layer, we measure the dependence of *V*_FMR_ on the bias current and temperature. The resulting data can then be compared with those obtained using other techniques. We first use the 3T Hanle measurement, carried out in a perpendicular field, to measure the spin accumulation at different injection bias currents^6^,^37^. Figure 3a shows the measured 3T signal *V*_3T_ at 30 K for different forward bias currents, and Fig. 3b shows *V*_3T_ at a fixed forward bias current for different temperatures. The asymmetric lineshape and the narrowing of the peak at low magnetic field in Fig. 3a are caused by the hyperfine interaction between the injected spins and the polarized nuclei^38^. At low temperature, *V*_3T_ is nearly proportional to the spin accumulation^6^, and so we expect the bias dependences of *V*_3T_ and *V*_FMR_ to be the same. In Fig. 3c, *V*_3T_ and the FMR signal at 30 K are plotted as a function of the bias voltage. The two measurement techniques agree up to a single bias-independent scale factor, which depends only on the average orientation of the magnetization in the FM relative to the spin accumulation in the SC. As discussed below, the FMR signal at small cone angles scales as the square of the angular precession amplitude in the FM. Unfortunately, this comparison cannot be made at high temperatures, at which a spin-dependent contribution to *V*_3T_ cannot be measured, as can be seen from the 300 K data in Fig. 3b. We have also performed non-local Hanle measurements^5^ on this sample, and a representative plot is shown in Supplementary Fig. 3. Although consistent with *V*_3T_ up to 120 K, the non-local Hanle signal also cannot be measured at higher temperatures.

The steady-state spin accumulation in the SC depends on the spin lifetime and diffusion constant^7^, which are strong functions of temperature. The Hanle techniques fail at high temperatures because the magnetic field scale corresponding to 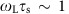 grows dramatically as *τ*_s_ decreases. As a result, the Hanle curves broaden and cannot be distinguished from field-dependent backgrounds. This limitation does not have an impact on the FMR signal, as we can see by comparison with non-local spin valve measurements^5^. Spin valves with source–detector separations between 250 nm and 5 μm were fabricated for each heterostructure by electron beam lithography. A micrograph of a spin valve device with a source–detector separation of 250 nm is shown in Supplementary Fig. 4. In non-local spin valve measurements, the field is swept in the plane, and the spin accumulation is inferred from the magnitude of the jump *V*_NLSV_ in the voltage when the source and detector magnetizations switch from parallel to antiparallel. Data for Co_2_FeSi/n-GaAs at a source–detector separation of 250 nm are shown in Fig. 3d. This spacing is smaller than the spin diffusion length of ∼800 nm at room temperature (Supplementary Fig. 5), and therefore it is possible to measure *V*_NLSV_ over the entire temperature range of this experiment. Figure 3e compares *V*_FMR_ with *V*_NLSV_ for both Co_2_FeSi/*n*-GaAs and Co_2_MnSi/*n*-GaAs from 30 to 300 K. The FMR signal sizes are normalized by the precession amplitude as discussed in Supplementary Note 2. The temperature dependences are similar for both sets of measurements on each heterostructure. Note that the choice of FM should not matter in this case, as the temperature dependence is governed primarily by properties of the SC.

### Modelling and measuring the frequency dependence

**Figure 4:**
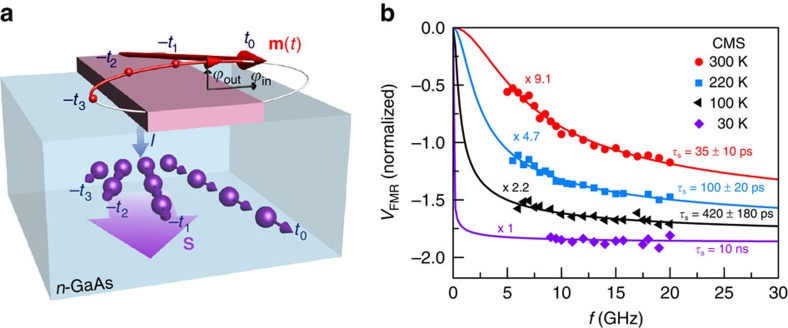
Spin accumulation in the short spin lifetime regime and frequency dependence of *V*_FMR_. (**a**) Injected spins (small purple arrows) in *n*-GaAs are parallel to the instantaneous orientation of the magnetization (red arrow on top of the FM) at the time they are injected. Earlier injected spins, for example, at *t*=−*t*_1_, −*t*_2_ or −*t*_3_ have progressively smaller magnitudes because of spin relaxation and diffusion. The orientation of the average spin accumulation, indicated by the large purple vector **S**, lags behind the magnetization **m**, which decreases 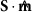 relative to the static case (*ω*=0). (**b**) *V*_FMR_, normalized by the precession amplitude, plotted as a function of frequency at different temperatures. For clarity, signal sizes are multiplied by the factors shown in the plot. The solid lines are fits based on equation (4) derived in the main text, from which the electron spin lifetime in the SC is obtained. For the 30 K data, the solid line is calculated from the expression of *V*_FMR_(*ω*) by using the known spin lifetime *τ*_s_=10 ns.

The data of Fig. 3 demonstrate that *V*_FMR_ is proportional to the spin accumulation. We now consider the frequency dependence of *V*_FMR_ and its relationship with *τ*_s_. Remarkably, we find that the FMR technique can provide a measurement of the spin lifetime in the regime 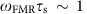, which turns out to be accessible at high temperatures. In this limit, a spin injected from the FM relaxes before the magnetization precesses through a complete cycle. This physical scenario is illustrated in Fig. 4a, in which the spin accumulation is represented by a series of spins (in purple) that have been injected into the SC at the times indicated, where *t*_0_ corresponds to the present, and −*t*_3_ through −*t*_1_ represent previous times as labelled on the trajectory of the precessing magnetization, which is shown in red. The spins shown are oriented approximately parallel to the magnetization at the time at which those spins were injected. Precession in the SC is ignored because 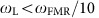 for the entire frequency and magnetic field range covered by the experiment. The smaller number of spins present from the earlier times is due to the relaxation of spins over time. The average spin accumulation, shown by the large purple arrow in Fig. 4a, is therefore a vector that lags behind the precessing magnetization.

The spin accumulation signal under these conditions can be calculated by an appropriately weighted time average of equation (1), incorporating spin relaxation and diffusion^6^:


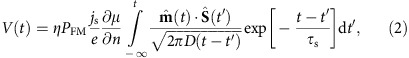


where *j*_s_ is the injected spin current density, while *D* and *τ*_s_ are the spin diffusion constant and the spin lifetime in *n*-GaAs. Here 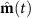 and 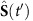 represent the time-dependent orientations of the magnetization and the injected spin polarization, respectively. The integral over *t*′ evaluates the projection of the spins injected at all previous times, reduced by the effects of diffusion and relaxation, on the present magnetization direction 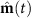. This is the instantaneous voltage detected by the FM. In the FM thin film, the precessing magnetization traces out an ellipse, with an in-plane cone angle *ϕ*_in_ and an out-of-plane angle *ϕ*_out_. From geometric analysis (ignoring the Larmor precession in the SC as noted in the previous paragraph), 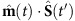 takes the form


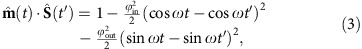


(see Supplementary Fig. 6 and Supplementary Note 4), in which *ω* is precession frequency in the FM. The d.c. voltage 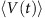 in the presence of a microwave field is the average value of *V*(*t*) over a precession period. The magnitude of *V*_FMR_(*ω*) is the difference of 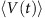 between resonance (*ω=ω*_FMR_) and off resonance, which can be determined by setting *ω*=0 in equation (2). We then obtain.


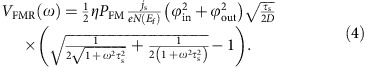


From equation (4) we obtain 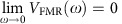. This corresponds to the d.c. limit, in which the orientation of the spin accumulation is always able to follow the magnetization. As *ω* increases, the angle by which the spin accumulation lags the magnetization increases, and *V*_FMR_(*ω*) becomes more negative. The expression for *V*_FMR_(*ω*) suggests that in the regime *ωτ*_s_∼1, *V*_FMR_(*ω*) will have considerable frequency dependence. This regime corresponds to short spin lifetimes, that is, *τ*_s_∼16 ps for a typical FMR frequency of *f*=ω/2π=10 GHz. As *ω* increases further, 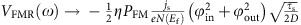 and saturates (no frequency dependence). This situation is equivalent to the long *τ*_s_ limit, which occurs at low temperature. We note that the magnitude of *V*_FMR_(*ω*) in this case is proportional to the steady-state spin accumulation, with the proportionality determined by the magnitude of the precession cone angles.

To test this model, we measure the frequency dependence of *V*_FMR_ at different temperatures. During the measurement, the injection bias current is fixed, and the amplitude of the magnetization precession, which is measured from the TAMR at reverse bias^29^, is fixed at a constant value for each temperature by adjusting the microwave power at each frequency (Supplementary Note 2). This procedure also accounts for the fact that the susceptibility of the FM (and hence the amplitude of magnetization precession for a given microwave power) depends on frequency (Supplementary Fig. 7). An additional and more subtle correction, discussed in Supplementary Note 3 and Supplementary Fig. 8, accounts for the differing in-plane and out-of-plane susceptibilities, the ratio of which also depends on frequency (Supplementary Fig. 9). Figure 4b presents the experimental results. The data are normalized by the magnetization precession amplitude, as discussed in Supplementary Note 2. The FMR signal shows a pronounced frequency dependence at high temperatures. The magnitude of the signal increases as the FMR frequency increases, and the sign of the effect is negative as expected from the decrease in 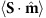. The solid lines in Fig. 4b are fits to *V*_FMR_(*ω*) given by equation (4), except for the 30-K data, for which *τ*_s_ is fixed at the value (10 ns) inferred from non-local spin valve measurements. Besides an overall scale factor, the spin lifetime *τ*_s_ is the only fitting parameter (*D* is absorbed into the prefactor in equation (4)). We obtain a spin lifetime of *τ*_s_=35±10 ps in *n*-GaAs at room temperature. This value is comparable to previous theoretical calculations based on D' yakonov and Perel' spin relaxation in bulk GaAs (ref. 39) as well as the value of *τ*_s_ (50±10 ps) extracted from non-local spin valve measurements shown in Supplementary Fig. 5. The frequency dependence in Fig. 4b disappears as the temperature is lowered to 30 K. In this limit, the magnetization precesses through multiple cycles before the spin accumulation relaxes, and *V*_FMR_ should therefore be insensitive to frequency, as is found experimentally.

## Discussion

In summary, we have demonstrated a technique based on FMR to measure spin accumulation in FM–SC devices. The precession of the magnetization at resonance leads to a measurable phase lag between the spin accumulation in the SC and the magnetization in the FM. Because the voltage detected by the FM is proportional to the projection of the spin accumulation on the magnetization, the spin accumulation voltage decreases at resonance. The typical FMR frequency is larger than the spin relaxation rate, and so the spin accumulation can be measured up to room temperature, making this approach more effective than the traditional Hanle effect, which has previously limited spin lifetime measurements in GaAs-based spin transport devices to temperatures less than ∼150 K. FMR occurs within a narrow magnetic field window, and the spin accumulation signal detected by the FM is sensitive to the precession of the magnetization. Together, these make the technique essentially immune to the field-dependent backgrounds that plague Hanle measurements, particularly at high temperatures. By measuring the frequency dependence of the spin accumulation signal, spin relaxation in the SC can be probed directly. With this method, we obtain an electron spin lifetime of a few tens of picoseconds in *n*-GaAs (*n*=3 × 10^16 ^cm^−3^) at room temperature. The FMR-based spin detection technique developed here can be used above room temperature and applied to other material systems, such as metals, in which the spin lifetimes are short and traditional Hanle measurements are impractical.

## Methods

### Sample growth

The FM/*n*-GaAs heterostructures investigated in this experiment were grown by molecular beam epitaxy on GaAs (001) substrates. The growth started with a 500 nm undoped GaAs buffer layer, followed by 2,500 nm of Si-doped *n*-GaAs (*n*=3 × 10^16 ^cm^−3^). The junction region consists of a 15 nm *n*→*n*^+^-GaAs transition layer followed by 18 nm *n*^+^ (5 × 10^18^ cm^−3^) GaAs (ref. 14). The 5-nm-thick FM film was then deposited epitaxially, followed by 10-nm thick Al and Au capping layers. The deposition temperatures for Co_2_MnSi and Co_2_FeSi were 220 and 270 °C, respectively.

### Device fabrication

Ion milling was used to define the 5 μm × 50 μm FM contacts. The remote 50 μm × 50 μm non-magnetic contacts were fabricated by depositing 40 nm Cu and then 40 nm Ge using electron beam evaporation. The Cu–Ge contacts^40^ were prepared in pairs and annealed by passing a current through them to make them ohmic. This step avoids thermal annealing of the FM/*n*-GaAs Schottky contact. A *n*-GaAs channel was defined by wet etching down to the substrate. SiN (80 nm) was deposited over the SC using plasma-enhanced chemical vapor deposition (PECVD) at a substrate temperature of 100 °C and then lifted off to expose the Cu–Ge and FM contacts. Finally, Ti/Au electrodes and bonding pads were fabricated by depositing 25 nm Ti and 120 nm Au using electron beam evaporation.

### Measurement

The FMR-spin detection measurement was carried out using a microwave generator, a lock-in amplifier and a current source, as shown in Fig. 1a. The microwave excitation signal was coupled to the FM injector through a coaxial cable and an ordinary wire bond. A 120-nm-thick Au wire patterned over the FM contact functioned as a stripline, which was terminated at ground. The d.c. current generating the spin accumulation was fed to the sample using a conventional twisted pair, with one lead connected to one of the ohmic contacts. The differential voltage between a second ohmic reference electrode and the FM contact (connected through a bias tee) was measured using a lock-in amplifier. The microwave signal was modulated using the 100-Hz reference signal of the lock-in amplifier, which was phased to the FMR signal. A background voltage due to rectification of the microwave current by the Schottky barrier leads to a field-independent background in both phases of the response; however, there is no FMR signal in the quadrature component. The circuit therefore measured the difference in the spin accumulation with and without microwave excitation. At each temperature, measurements were carried out in the power regime where the FMR response was linear with respect to the microwave field amplitude.

## Additional information

**How to cite this article:** Liu, C. *et al*. Dynamic detection of electron spin accumulation in ferromagnet–semiconductor devices by ferromagnetic resonance. *Nat. Commun.* 7:10296 doi: 10.1038/ncomms10296 (2016).

## Supplementary Material

Supplementary Information Supplementary Figures 1-9, Supplementary Notes 1-4 and Supplementary References
