# Supplementary material for: Dynamic detection of electron spin accumulation in ferromagnet–semiconductor devices by ferromagnetic resonance
Source: Nat Commun. 2016 Jan 18;7:10296. doi: 10.1038/ncomms10296 (PMC4735623; doi:10.1038/ncomms10296)
Supplement: Supplementary Information — Supplementary Figures 1-9, Supplementary Notes 1-4 and Supplementary References [file ncomms10296-s1.pdf]

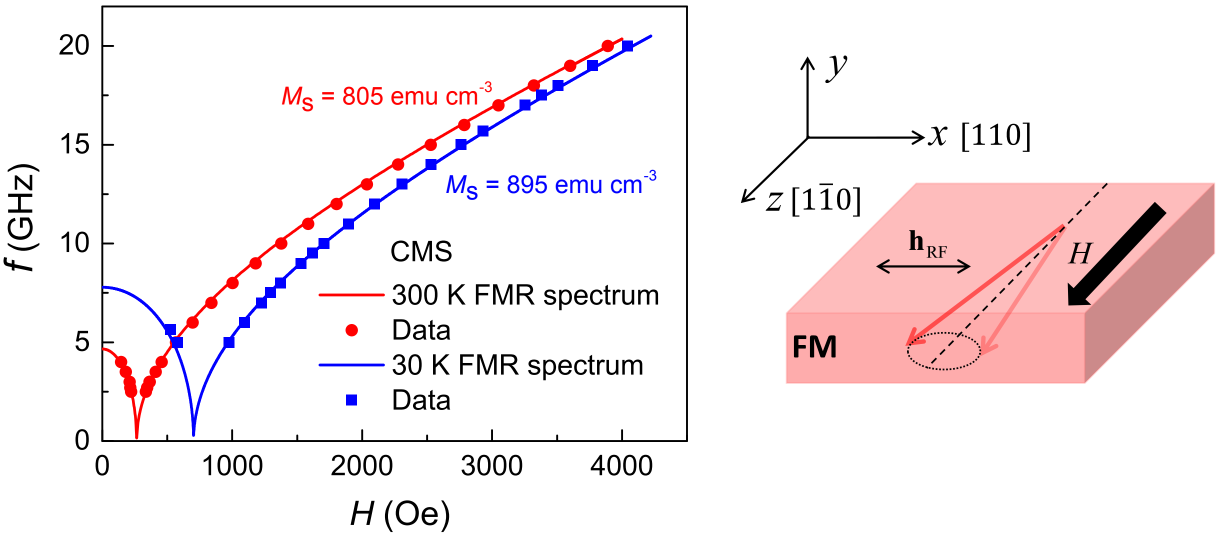

Supplementary Figure 1. Resonant fields for each microwave frequency (closed symbols) and the calculated FMR spectrum based on Supplementary Equation 1 (solid lines) for the Co_2MnSi/*n*-GaAs sample. The drawing on the right illustrates the measurement geometry. The magnetic field is applied along the in-plane magnetic hard-axis. At low frequencies, there are two branches in the FMR spectrum. The lower branch corresponds to the case when the magnetization is not aligned with the applied field. The data discussed in this paper were obtained for magnetic fields above saturation and for frequencies for which there is only a single branch.

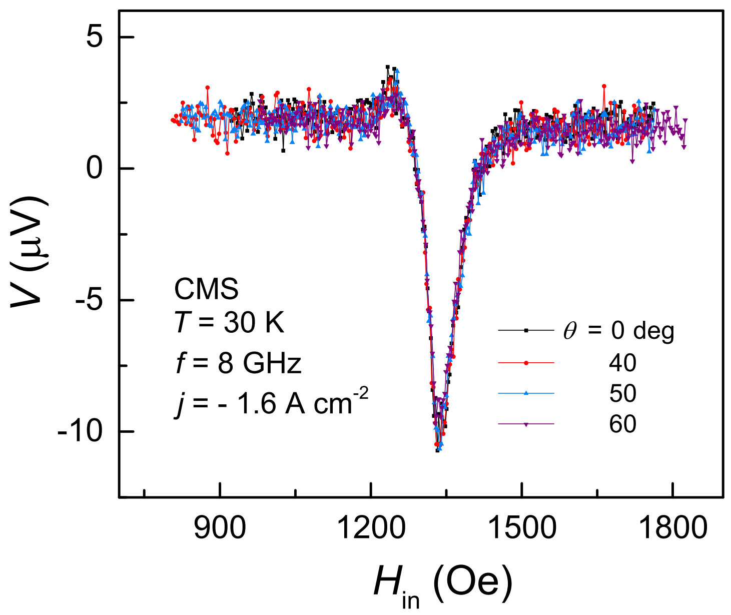

Supplementary Figure 2. Data in Fig. 2c of the main text plotted as a function of the in-plane component of the total magnetic field. The linewidths of the resulting FMR curves are nearly the same. This demonstrates that the increase in the FMR linewidth is associated only with the geometry of the applied field.

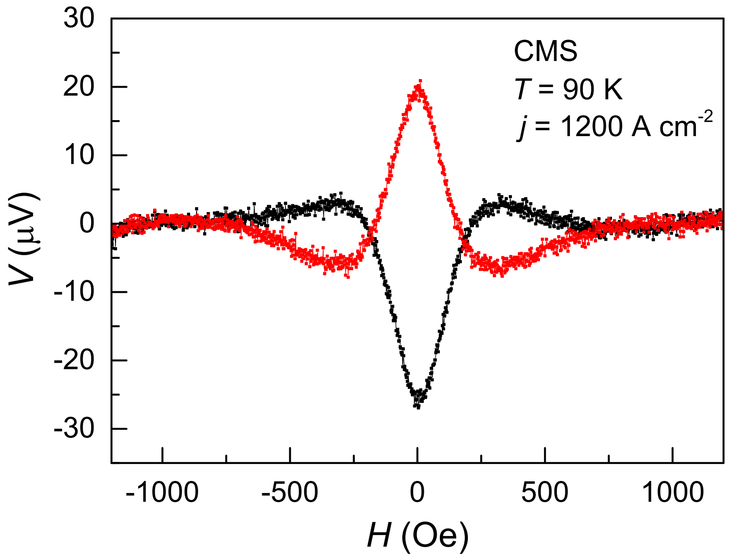

Supplementary Figure 3. Non-local Hanle measurement on a Co_2MnSi/n-GaAs sample at 90 K. Red and black data correspond to anti-parallel and parallel configurations for the magnetization, respectively.

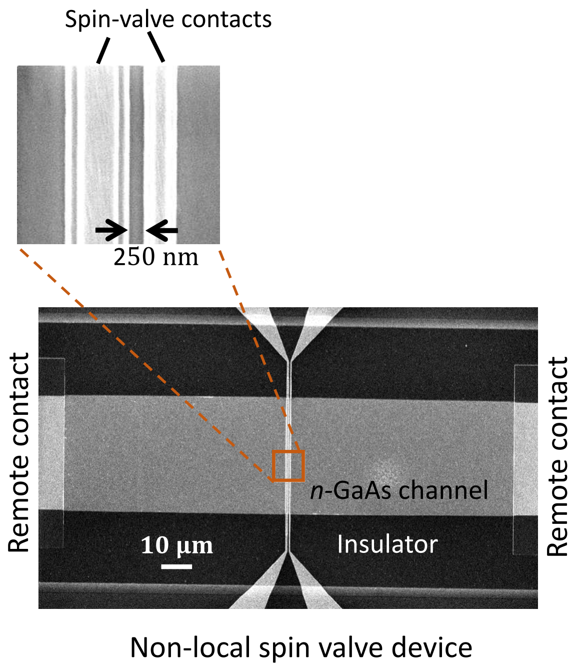

Supplementary Figure 4. Micrograph of a non-local spin valve device fabricated using electron beam lithography.

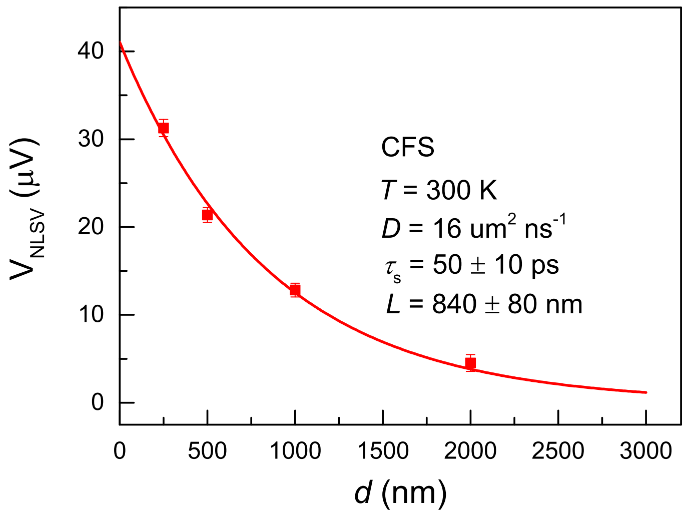

Supplementary Figure 5. Non-local spin valve signal measured as a function of the separation between the spin injector and detector. D, τ_s and L shown in the legend are the spin diffusion constant, lifetime and diffusion length, respectively. An exponential fit to the data gives a spin diffusion length of about 800 nm at room temperature. The spin lifetime τ_s extracted from this measurement is 50 ± 10 ps.

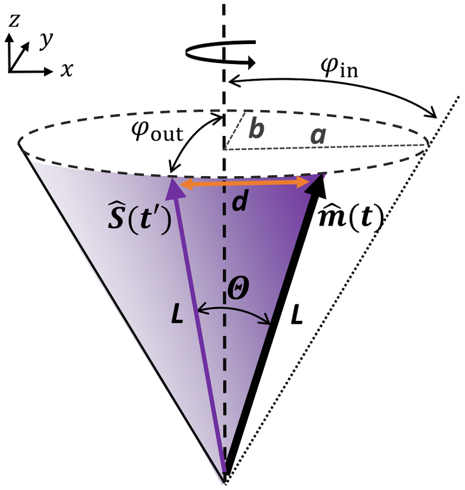

Supplementary Figure 6. Geometrical relationship between $\hat{\mathbf{m}}(t)$ and $\hat{\mathbf{S}}(t')$. The purple color distributed around the cone represents previously injected spins, with the most recent spins shown in the darkest color. Because we are ignoring precession in the SC, the orientation of the injected spins is determined entirely by the orientation of $\hat{\mathbf{m}}$ at the instant they were injected. The unit vector $\hat{\mathbf{S}}(t')$ is parallel to the instantaneous injected spin polarization at t', where $t' \le t$. The distances a, b and L are dimensionless. They are related to the in-plane and out-of-plane cone angles by $\sin\varphi_{\text{in}} = \frac{a}{L}$ and $\sin\varphi_{\text{out}} = \frac{b}{L}$. Note that the FM film lies in the x, z plane.

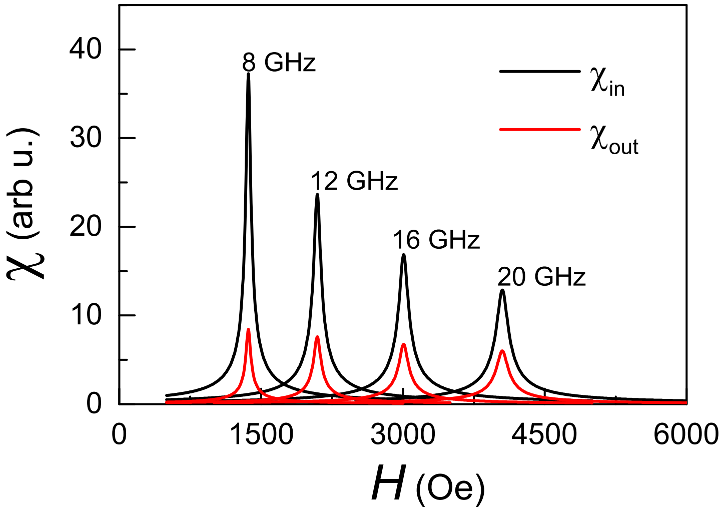

Supplementary Figure 7. Dynamical susceptibilities calculated as a function of the magnetic field at different frequencies. χ_{in} (black) and χ_{out} (red) are the in-plane and out-of-plane components of the susceptibilities, respectively.

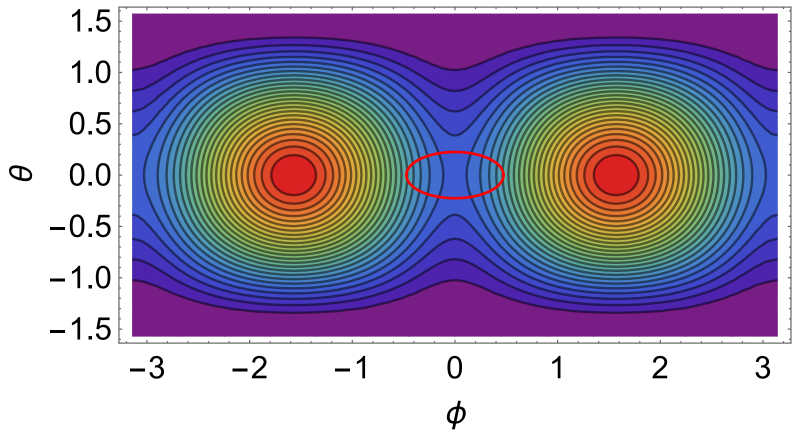

Supplementary Figure 8. Contour plot showing the measured tunneling anisotropic magnetoresistance (TAMR) generated voltage as a function of the orientation of the magnetization[1]. ϕ and θ are the in-plane and out-of-plane angles of the magnetization. The origin $(\phi = 0, \theta = 0)$ corresponds to the in-plane $[1\bar{1}0]$ direction. The red ellipse illustrates the trajectory of the precessing magnetization at FMR. The precession of the magnetization causes a change in voltage as shown by the contours in the plot. The change in voltage is much weaker in the θ direction than that in the ϕ direction.

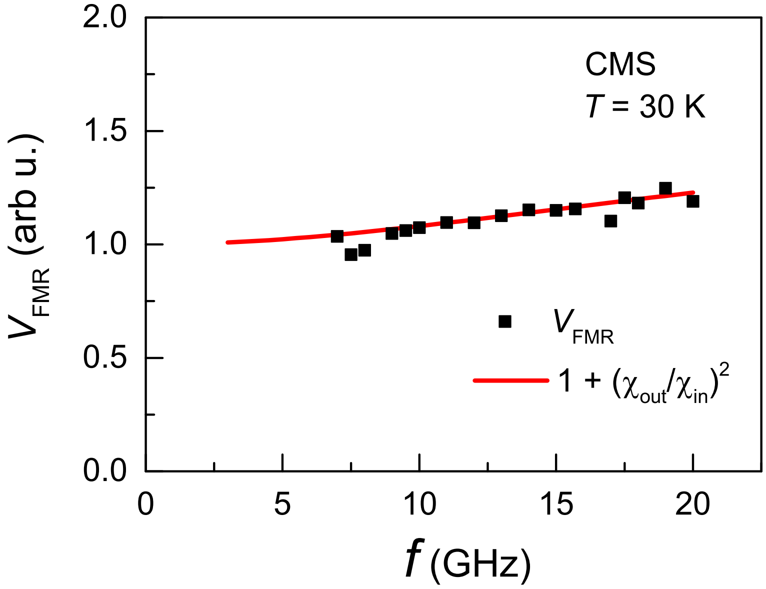

Supplementary Figure 9. FMR signal measured at 30 K as a function of frequency. The solid line is $1+\frac{\varphi_{\rm out}^2}{\varphi_{\rm in}^2}$ plotted as a function of FMR frequency. For comparison purposes, the sign of the FMR signal is reversed, and a scale factor is applied to bring the value of the 7 GHz data point to 1.

Supplementary Note 1: The FMR spectrum

In this experiment, the dc magnetic field is applied along the in-plane magnetic hard-axis of the ferromagnet (FM), which is also the $[1\bar{1}0]$ crystal axis and the z-axis in Supplementary Fig. 1. The Oersted field generated by the microwave stripline is along the $[110]$ crystalline axis, which is the x-axis in Supplementary Fig. 1. The upper branch (high field part) of the FMR spectrum corresponds to the case when the magnetization is saturated. The resonance frequency can be calculated by using Kittel's formula

$$f = \frac{\gamma}{2\pi}\sqrt{(H + N_{\mathrm{x}}M_{\mathrm{s}})(H + N_{\mathrm{y}}M_{\mathrm{s}})}, \tag{1}$$

where γ is the gyromagnetic ratio in the FM, and N_{x} and N_{y} are the effective demagnetization factors in the in-plane transverse (x) and out-of-plane (y) directions. Note that the effective demagnetization factors account for both the shape and crystalline anisotropies. Because the film is only 5 nm thick, we assume $N_{\mathrm{y}} = 4\pi$. M_{s} is the saturation magnetization, which is obtained by measuring the saturation field H_{out} in the out-of-plane direction and using $M_{\mathrm{s}} = \frac{H_{\mathrm{out}}}{4\pi}$. In these Heusler samples, the in-plane anisotropy is dominated by the uniaxial surface anisotropy, and so N_{x} can be determined by measuring the saturation field H_{in} along the in-plane hard axis direction and using $N_{\mathrm{x}} = -\frac{H_{\mathrm{in}}}{M_{\mathrm{s}}}$.

The lower branch (low field part) of the FMR spectrum corresponds to the case when the magnetization is not aligned with the applied magnetic field. Assuming coherent rotation, the equilibrium condition for the magnetization is $\cos\phi = \frac{HM_{\mathrm{s}}}{2K_{\mathrm{u}}}$, where $K_{\mathrm{u}} = \frac{H_{\mathrm{in}}M_{\mathrm{s}}}{2}$ is the in-plane uniaxial anisotropy energy. To apply Supplementary Equation (1), H is replaced by the component parallel to the precession axis (equilibrium orientation of the magnetization), while $N_{\mathrm{y}} = 4\pi$ and $N_{\mathrm{x}} = -\frac{2K_{\mathrm{u}}}{M_{\mathrm{s}}^2}\cos 2\phi$.[2] We note that all of the parameters required to calculate the FMR frequency are measured independently, and the calculated branches of the spectrum are shown as solid lines in Supplementary Fig. 1. The data in this paper were obtained at frequencies where there is only a single branch and the magnetization is saturated (above 5 GHz at 300 K and 8 GHz at 30 K).

Supplementary Note 2: Calibrating the FMR precession cone angle using the TAMR effect

In these epitaxially grown FM/n-GaAs heterostructures, a Schottky tunnel barrier forms at the FM/n-GaAs interface. It has been found that the tunneling resistance across the FM/n-GaAs interface depends on the orientation of the magnetization relative to the crystal axes[3,4], which is known as the tunneling anisotropic magnetoresistance (TAMR) effect. In previous work, we

found that the TAMR leads to a dc voltage peak at FMR. The magnitude of the dc voltage peak is proportional to a weighted sum of the squares of the precession cone angles[1]

$$V_{\mathrm{FMR-TAMR}} = \frac{1}{2}\triangle V_{\mathrm{TAMR}}\varphi_{\mathrm{in}}^2 - \frac{1}{2}\triangle V_{\mathrm{TAMR-out}}\varphi_{\mathrm{out}}^2, \tag{2}$$

where $V_{\mathrm{FMR-TAMR}}$ denotes the FMR signal produced by the TAMR effect, and φ_{in} and φ_{out} are the in-plane and out-of-plane precession cone angles of the magnetization at FMR. The ratio $\varphi_{\mathrm{out}}/\varphi_{\mathrm{in}}$ can be calculated by using the measured magnetic anisotropies of the FM film. Typically, φ_{out} is much smaller than φ_{in} at low frequencies due to the shape anisotropy of the FM thin film. $\triangle V_{\mathrm{TAMR}}$ and $\triangle V_{\mathrm{TAMR-out}}$ are the change in the interface voltage caused by the TAMR effect. They are measured by applying a fixed bias current and measuring the dc voltage as the magnetization is rotated by 90 degrees in the sample plane and in the out-of-plane direction, respectively. Given this information, φ_{in} and φ_{out} at a given microwave power can be determined from $V_{\mathrm{FMR-TAMR}}$.

The actual amplitudes of the experimental precession cone angles at FMR are a function of temperature because both the Gilbert damping of the FM and the microwave coupling to the sample are temperature dependent. We ensure that this does not impact the analysis by carrying out the FMR measurement at both reverse and high forward bias. In these samples, there is no spin injection when the FM/n-GaAs interface is reverse-biased, and the entire FMR voltage signal is therefore due to $\triangle V_{\mathrm{TAMR}}$. According to Supplementary Equation (2), the magnitude of the FMR signal associated with the TAMR effect determines φ_{in} and φ_{out}. When a large forward bias current ($j \geqslant 400$ A cm^{-2}) is applied across the FM/n-GaAs interface, the FMR signal is dominated by the spin accumulation in the n-GaAs. The spin signal presented in Fig. 3e of the main text is then normalized as

$$V_{\mathrm{FMR}}(\mathrm{normalized}) = \frac{V_{\mathrm{FMR-spin}}}{V_{\mathrm{FMR-TAMR}}}\triangle V_{\mathrm{TAMR}}, \tag{3}$$

where $V_{\mathrm{FMR-spin}}$ is the raw magnitude of the FMR signal produced by the spin accumulation.

Supplementary Note 3: Dynamical susceptibilities and the effects of the shape of the precession ellipse

In taking the frequency dependence measurement, we adjust the microwave power at each frequency so that the amplitude of the FMR peak produced by the TAMR effect at a fixed reverse bias is fixed. By doing this, we can control the precession cone angle over the entire frequency range. From Supplementary Fig. 8, we see that the measured TAMR voltage has little dependence on the out-of-plane angle θ of the magnetization ($\triangle V_{\mathrm{TAMR-out}}$ is small). Therefore, the FMR signal produced by the TAMR effect depends only on φ_{in}^2 (see Supplementary Equation (2)). However, the

shape of the ellipse traced out by the magnetization changes as the FMR frequency increases. The magnitudes of the in-plane and out-of-plane components of the magnetization driven by the microwave magnetic field are given by $m_{\rm in} = h_{\rm RF}\chi_{\rm in}$ and $m_{\rm out} = h_{\rm RF}\chi_{\rm out}$, respectively. The dynamical susceptibilities of our samples can be derived as:

$$\chi_{\rm in} = \frac{\gamma^2(H_0 + 4\pi M_{\rm s})M_{\rm s}}{\omega_0^2 - \omega^2}, \tag{4}$$

$$\chi_{\rm out} = \frac{-i\omega\gamma M_{\rm s}}{\omega_0^2 - \omega^2}, \tag{5}$$

$$\omega_0^2 = [\gamma(H_0 + 4\pi M_{\rm s}) + i\alpha\omega][\gamma(H_0 + N_{\rm x}M_{\rm s}) + i\alpha\omega]. \tag{6}$$

H_0 is the total dc magnetic field parallel to the precession axis and ω is the microwave frequency. In the expression for ω_0^2, α is the Gilbert damping, and the in-plane surface anisotropy induced effective demagnetization factor $N_x \approx -0.80$ at $T = 30$ K. For the case when an oblique magnetic field is applied, we first calculate the equilibrium orientation of the magnetization, and then calculate the effective demagnetization factors in that equilibrium orientation. In the measurements shown in Fig. 2 of the main text, when $\theta = 60°$, the magnetization tilts into the out-of-plane direction by about 10 degrees at the resonance field of 2525 Oe. Therefore in Supplementary Equation (4) , $N_{\rm y} = 4\pi \approx 12.6 \rightarrow N_{\rm y'} = 11.8$, which reduces the susceptibility $\chi_{\rm in}$ by about 6%. This effect, as seen in Fig. 2c of the main text, is small compared to the suppression of the FMR signal due to the Hanle effect in the semiconductor at 30 K (Figs. 2a and 2d of the main text).

The relative magnitudes of the dynamical susceptibilities $\chi_{\rm in}$ and $\chi_{\rm out}$ change as the FMR frequency increases. Supplementary Fig. 7 shows the susceptibilities $\chi_{\rm in}$ and $\chi_{\rm out}$ plotted as a function of the magnetic field at different frequencies. As shown in the plot, the magnitude of $\chi_{\rm out}$ relative to $\chi_{\rm in}$ increases with frequency. This causes the shape of the ellipse to become more circular as the FMR frequency increases.

According to equation (4) in the main text, $V_{\rm FMR}(\omega)$ is proportional to the sum of the squares of the in-plane and out-of-plane cone angles

$$V_{\rm FMR}(\omega) \propto (\varphi_{\rm in}^2 + \varphi_{\rm out}^2) = \varphi_{\rm in}^2(1 + \frac{\varphi_{\rm out}^2}{\varphi_{\rm in}^2}). \tag{7}$$

At a given microwave power, $\varphi_{\rm in}$ and $\varphi_{\rm out}$ are proportional to $\chi_{\rm in}$ and $\chi_{\rm out}$, respectively. Therefore, for a fixed $\varphi_{\rm in}^2$, which is set to a fixed value using the TAMR calibration, $V_{\rm FMR}(\omega)$ increases as the ratio $\frac{\chi_{\rm out}}{\chi_{\rm in}}$ increases. In the frequency dependence measurement at 30 K, a slight increase in the FMR signal is observed as the FMR frequency increases. Supplementary Fig. 9 shows the magnitude of the spin accumulation measured by FMR as a function of frequency at 30 K. The slight increase in the signal size with frequency in this figure matches the calculation for the change in $1+\frac{\varphi_{\rm out}^2}{\varphi_{\rm in}^2}$, which is shown by the solid line. In the main text, we have corrected for this effect in the data shown in Fig. 4b, so that the change in the signal size as a function of the FMR frequency is due only to the spin relaxation in n-GaAs.

Supplementary Note 4: Finding $\hat{\mathbf{m}}(t)\cdot\hat{\mathbf{S}}(t')$

In Supplementary Fig. 6 the coordinate system is defined such that the z axis is along the FMR precession axis, and the x and y axes are along the long (in-plane) and short (out-of-plane) axes of the ellipse traced out by the magnetization. The semi-major and semi-minor axes are labeled as a and b, respectively. Θ is the angle between the vectors $\hat{\mathbf{m}}(t)$ and $\hat{\mathbf{S}}(t')$. Using trigonometry,

$$\cos\Theta = \frac{L^2+L^2-d^2}{2L^2}, \tag{8}$$

$$d^2 = (X_{\rm m}-X_{\rm s})^2+(Y_{\rm m}-Y_{\rm s})^2. \tag{9}$$

In Supplementary Equation (8) , $X_{\rm m}$ and $Y_{\rm m}$ are the x and y coordinates of $\hat{\mathbf{m}}(t)$ on the precession ellipse, while $X_{\rm s}$ and $Y_{\rm s}$ are the x and y coordinates of $\hat{\mathbf{S}}(t)$ on the same ellipse. When the magnetization is precessing at FMR, $X_{\rm m}=a\cos\omega t$, $Y_{\rm m}=b\sin\omega t$, $X_{\rm s}=a\cos\omega t'$ and $Y_{\rm s}=b\sin\omega t'$. Substituting these expressions into Supplementary Equation (8) , we obtain

$$\cos\Theta = 1-\frac{a^2}{2L^2}(\cos\omega t-\cos\omega t')^2-\frac{b^2}{2L^2}(\sin\omega t-\sin\omega t')^2. \tag{10}$$

From Supplementary Fig. 6, $\frac{a}{L}=\sin\varphi_{\rm in}\approx\varphi_{\rm in}$ and $\frac{b}{L}=\sin\varphi_{\rm out}\approx\varphi_{\rm out}$. These approximations hold because the actual precession cone angles are small. Finally we obtain

$$\cos\Theta = 1-\frac{\varphi_{\rm in}^2}{2}(\cos\omega t-\cos\omega t')^2-\frac{\varphi_{\rm out}^2}{2}(\sin\omega t-\sin\omega t')^2. \tag{11}$$

---

1. Liu, C. *et al.* Electrical detection of ferromagnetic resonance in ferromagnet/$n-$GaAs heterostructures by tunneling anisotropic magnetoresistance. *Appl. Phys. Lett.* **105**, 212401 (2014).
2. Kittel, C. On the theory of ferromagnetic resonance absorption. *Phys. Rev.* **73**, 155–161 (1948).

3. Moser, J. *et al.* Tunneling anisotropic magnetoresistance and spin-orbit coupling in Fe/GaAs/Au tunnel junctions. *Phys. Rev. Lett.* **99**, 056601 (2007).
4. Matos-Abiague, A., Gmitra, M. & Fabian, J. Angular dependence of the tunneling anisotropic magnetoresistance in magnetic tunnel junctions. *Phys. Rev. B* **80**, 045312 (2009).
